# External pressure dynamics promote kidney viability and perfusate filtration during ex vivo kidney perfusion

**DOI:** 10.1038/s41598-022-26147-5

**Published:** 2022-12-13

**Authors:** Yuhei Higashi, Jun Homma, Hidekazu Sekine, Hiroki Yago, Eiji Kobayashi, Tatsuya Shimizu

**Affiliations:** 1 Institute of Advanced Biomedical Engineering and Science, TWIns, Tokyo Women’s Medical University, 8-1 Kawada-Cho, Shinjuku-Ku, Tokyo, 162-8666 Japan; 2 Department of Kidney Regenerative Medicine, Industry-Academia Collaborative Department, The Jikei University School of Medicine, Tokyo, Japan; 3 Tokaihit Co., Ltd., Shizuoka, Japan

**Keywords:** Biotechnology, Health care, Medical research

## Abstract

Normothermic machine perfusion (NMP) has not yet been established as a technique for preserving organs for a day. A key contributing factor to the same is that the perfusing solutions cannot circulate continuously and evenly in the organs. Here, we conceived a method of applying intermittent air pressure from outside the organ to assist its circulatory distribution during perfusion. We used a perfusion culture system while applying external pressure to culture rat kidneys and compared the circulatory distribution in the kidneys, changes in tissue morphology due to injury, and perfusate filtration. The intermittent pressurization (IMP) (−) group showed markedly poorer circulation on the upper side compared with that in the lower side, alongside histological damage. On the other hand, the IMP (+) group showed improved circulation in the upper side and had lesser histological damage. Furthermore, the IMP (+) group maintained the ability to filter perfusate for 24 h. In transplantation medicine and regenerative medicine research, this method has the potential to contribute to more efficient organ preservation and more functional tissue regeneration in the future.

## Introduction

Organ transplantation is a treatment that replaces failed vital organs with healthy organs to restore function. Because ATP reduction, oxidative stress, and other disturbances in donor organs before delivery to the recipient can adversely affect organ viability after transplantation, organ preservation is important in transplantation medicine. There are two methods for preserving organs: immersion cooling and NMP. The immersion cooling preservation method is now commonly used in clinical practice because of the simplicity of the procedure and transportation. The permissible ischemia time before restarting blood flow once the organ has been harvested from the donor is 4 h for the heart^1^, 8 h for the lungs^2^, 12 h for the liver and small intestine^3,4^, and 24 h for the pancreas and kidneys^5,6^. Organ transplants from marginal donors, such as cardiac arrest donors or elderly donors, have gradually become mainstream in the recent years to increase the number of donor organs. However, immersion cooling preservation has been shown to induce considerable loss of organ structure and physiologic function after transplantation, resulting in poor prognosis^7–10^. Organs preserved via immersion cooling are ischemic. After transplantation, blood flows at once into the ischemic organ, producing a variety of toxic substances and causing ischemia–reperfusion injury.

On the other hand, NMP supports cellular metabolism by allowing for the delivery of oxygen and nutrients, as well as the elimination of waste products inside the cells. This method has been shown to reduce ischemia–reperfusion injury after transplantation and increase organ viability by avoiding ischemic conditions, compared with immersion cooling^11–14^. Perfusion at physiological temperatures is a method that aims to achieve normal metabolism while avoiding damage caused by low temperatures. This method is expected to immediately restore organ function after transplantation and increase organ viability, compared with the hypothermic perfusion method; therefore, it has been investigated in a variety of organs^15–17^. Nasralla et al.^16^ used this method to evaluate deviations in enzyme, ATP, and lactate levels during 12-h liver preservation and reported a reduced number of discarded donor organs and early functional recovery after transplantation. Progress in organ preservation through perfusion at physiological temperatures may further extend preservation time and lower the risk of functional failure after transplantation in the future.

However, it is difficult to perfectly reproduce circulation in vitro with NMP, because the circulation of the perfusing solutions is controlled only by a pump. Notably, the circulatory distribution that changes with multiple feedback adjustments, such as cardiac output and vascular resistance, is difficult to reproduce. There are two methods for delivering perfusing solutions, steady flow and pulsatile flow. Steady flow further has two methods, controlled constant arterial pressure and controlled constant perfusion rate^18,19^. The former has the advantage of preventing blood vessel breakdown caused by a sudden increase in arterial pressure during stenosis or occlusion. However, reduced flow and interrupted perfusion due to the presence of micro-clots due to poor perfusion during washout the accumulation of cellular debris shed during injury might disrupt circulation to the periphery if untreated. The latter can keep the whole organ perfused, but accumulation of cellular debris can raise intravascular pressure and cause the blood vessels to break down over time. Thus, when organs are perfused in vitro, perfusion techniques are required to ensure that the perfusing solutions are circulated continuously and evenly throughout the organ. The upper side of small intestinal tissue pumped at a constant flow rate exhibited markedly worse circulation than the lower side^20^. A circulatory imbalance within the kidney has also been reported in kidneys pumped at a constant pressure^21,22^. Thus, in vitro steady flow makes perfusate delivery to the whole tissue challenging.

When an isolated organ is perfused via artificial circulation, pulsatile flow is a major factor in maintaining adequate peripheral circulation. Particularly in the kidneys, it has been reported that pulsatile flow maintains intrarenal circulation volume and reduces renal dysfunction, compared to a steady flow^23^. Gallinat et al.^24^ demonstrated that pulsatile flow improves sodium reabsorption and creatinine clearance by increasing vasorelaxant NO and decreasing vasoconstrictor ET-1, compared with steady flow. However, pulsatile flow generated by a pump increases the inner pressure of the blood vessel and expands its lumen due to the increased flow volume. Therefore, excessive pressure stress may damage vascular endothelial cells and disrupt the vascular structure. In addition, when the difference between internal and external pressure rises as a result of an increase in intravascular pressure, the perfusing solutions leak from the ruptured regions of the vascular structure. Furthermore, when the lumen of a vessel is blocked, the blockage is difficult to open using only the upstream pulsatile flow generated by the pump, which may be unable to circulate the perfusate uniformly in the organ.

Therefore, we conceived a method of applying intermittent air pressure from outside the organ to assist circulatory distribution during perfusion. This method can prevent the vascular structure breakdown caused by pressure stress because it increases the internal pressure of the blood vessel without expanding its lumen as a result of applying external pressure. In addition, the external pressure suppresses perfusing solution leakage from the ruptured area by reducing the difference between the intravascular and external pressures. Furthermore, the lumen diameter of the blood vessel at the front and back of the occluded area can be changed by applying external pressure to the whole organ in addition to the perfusion pressure from upstream, which is expected to reduce the risk of blockage in a blood vessel. This principle is also used to prevent venous thrombosis in the deep lower extremities as an intermittent pneumatic compression^25,26^. Positive pressure acts on the entire vascular system and increases circulation by changing the cross-sectional area of the blood vessels^27^. By increasing the systolic perfusion pressure of blood vessels in the range of 80–120 mmHg and in vivo biological blood pressure, we devised a method for applying external pressure to generate a pulsatile flow that mimics blood pressure changes in vivo. External pressure was applied intermittently to create a pulsatile flow in this experiment instead of varying the flow rate to maintain a constant pressure. Furthermore, while the previous system brought in air from the outside^20^, the new system used a roller pump that could rotate in both forward and reverse directions to apply external pressure by moving air between two sealed rooms.

In this study, we used an intermittent pressurization (IMP) system for perfusion culture of rat kidneys at physiological temperature, and we verified its usefulness by examining circulatory distribution in the kidneys, changes in tissue morphology due to injury, and perfusate filtration.

## Results

### Characteristics of the new pressurization system

**Figure 1:**
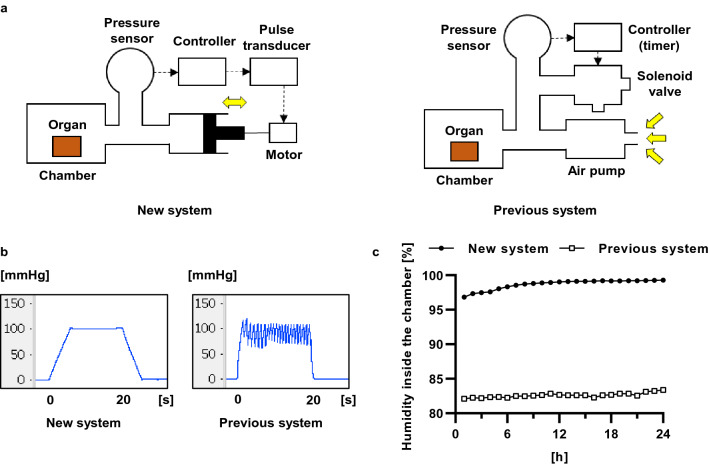
System for intermittent pressurization. (**a**) Schematic representation of the mechanism for intermittent pressurization. The new system operates by moving air inside an enclosed space. The previous system operated by pumping air from the outside. A sensor provides constant pressure feedback inside the chamber, pressurizing and depressurizing according to the set time. (**b**) Changes in chamber pressure at + 100 mmHg for 20 s of pressurization and depressurization using new and previous devices. (**c**) Changes in humidity inside the chamber for 24 h using new and previous devices. A sensor set inside the chamber maintained 37 °C. The data are shown as means (n = 3).

**Figure 2:**
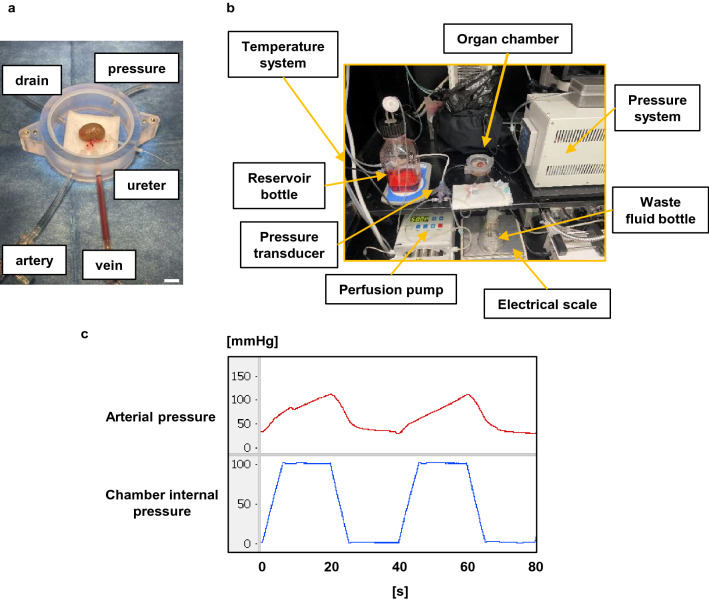
Normothermic machine perfusion system with an intermittent pressurization system for kidney perfusion culture. (**a**) The custom-made sealed chamber was used for perfusion of the left rat kidney. Five ports are provided to connect to the artery, vein, ureter, intermittent pressurization system, and waste fluid bottle. (**b**) The custom-made machine perfusion system. The system consisted of a perfusion pump, organ chamber, pressure system, temperature system, reservoir bottle, pressure transducer, waste fluid bottle, and electrical scale. The system set in the box maintained 37 °C. (**c**) Changes in chamber pressure and kidney arterial inlet pressure. A pressure of + 100 mmHg was applied intermittently for 20 s to the chamber.

The mechanism for pressurizing the chamber sent air back and forth between the chamber and the sealed container (Fig. 1a). The new system accurately applied an external pressure of + 100 mmHg (Fig. 1b). The humidity inside the chamber during the 24-h IMP revealed that the new system maintained a higher humidity than the previous system (new system: 98.69% vs. previous system: 82.60%; Fig. 1c). The left kidney with arteriovenous was implanted in a custom-made sealed chamber (Fig. 2a) and perfused from the artery using the normothermic machine perfusion system (Fig. 2b). In addition, the arterial pressures associated with increasing pressure in the chamber by intermittently applying a + 100 mmHg external pressure to the kidney during perfusion ranged from 80 to 120 mmHg, the systolic blood pressure in vivo (Fig. 2c). These results showed that kidney perfusion could be performed to mimic biological blood pressure using the constructed external pressure application system.

### Intrarenal circulation during short-term perfusion

**Figure 3:**
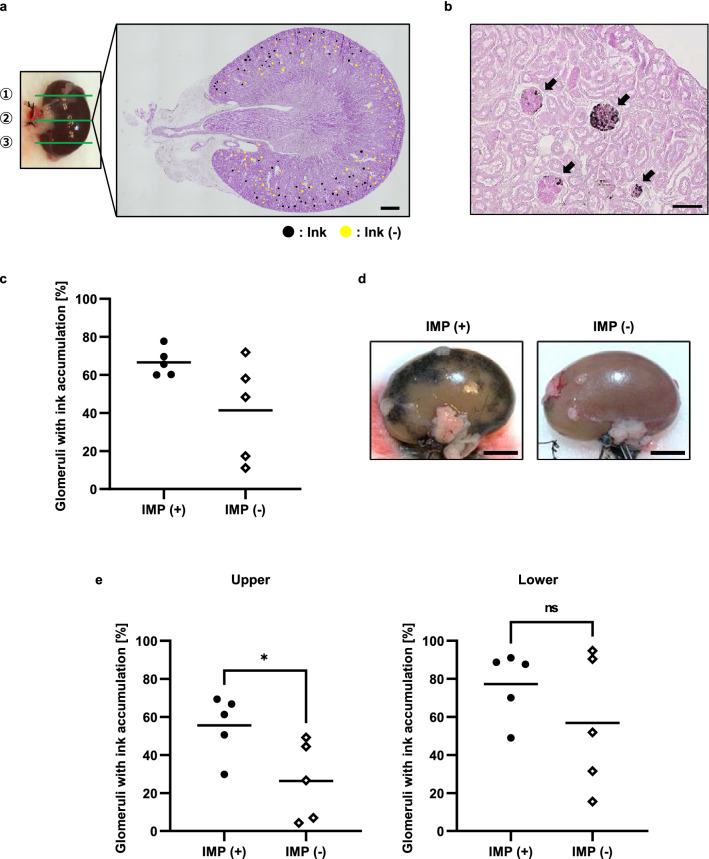
Short-term effect of intermittent pressurization on kidney perfusion. (**a**) Stained section for analysis of the intrarenal perfusion region. The green boxes indicate the three analyzed sites. Black dot: glomeruli with ink accumulation. Yellow dot: glomeruli without ink. Scale bars: 1 mm. (**b**) The stained image after ink perfusion. Black arrows: glomeruli with ink accumulation. Scale bars: 100 μm. (**c**) Point diagram comparing the percentage of accumulated glomerular counts after ink perfusion in the IMP (+) and IMP (−) groups. Fisher’s exact test. *p* < 0.05, n = 5. (**d**) The upper side of the kidney during ink perfusion in the IMP (+) and IMP (−) groups. Scale bars: 5 mm. (**e**) Comparing the percentage of glomeruli with ink accumulation on the upper side and lower side in the IMP (+) and IMP (−) groups. *p < 0.05, n = 5. *ns* not significant.

The number of glomeruli that accumulated ink was counted to evaluate the kidney perfusion region. The glomeruli that had accumulated ink were labeled in black, while those that did not were labeled in yellow (Fig. 3a). The ink particles accumulated inside the glomerular structure, as indicated by the arrows in Fig. 3b. The mean percentage of glomeruli with ink accumulation in the three sections revealed a marked difference between the samples in the IMP (−) group. In comparison with the IMP (−) group, all samples in the IMP (+) group had a mean percentage of glomeruli with ink accumulation > 60%, and the variation between samples was significantly reduced (IMP (+): 66.62% vs. IMP (−): 41.34%, F test: *P* < 0.05; Fig. 3c). The findings also revealed that IMP during short-term perfusion did not result in perfect kidney circulation.

Ink traces after perfusion were rarely detected in the upper side of kidney tissues in the IMP (−) group. In the IMP (+) group, however, ink accumulation increased in the upper side of the kidney tissue during perfusion (Fig. 3d). When the percentage of glomeruli with ink accumulation on the upper and lower sides were compared, both groups showed that ink tended to accumulate in the lower glomeruli (IMP (+): 77.30% vs. IMP (−): 56.81%; Fig. 3e lower). On the other hand, the number of glomeruli with ink accumulation on the upper side demonstrated that the IMP (+) group was generally superior to the IMP (−) group (IMP (+): 55.56% vs. IMP (−): 26.31%, *P* < 0.05; Fig. 3e upper). These results revealed that IMP during perfusion helped maintain uniform circulation in the kidney by reducing variation in circulation between specimens.

### Twenty-four-hour perfusion culture outcomes

**Figure 4:**
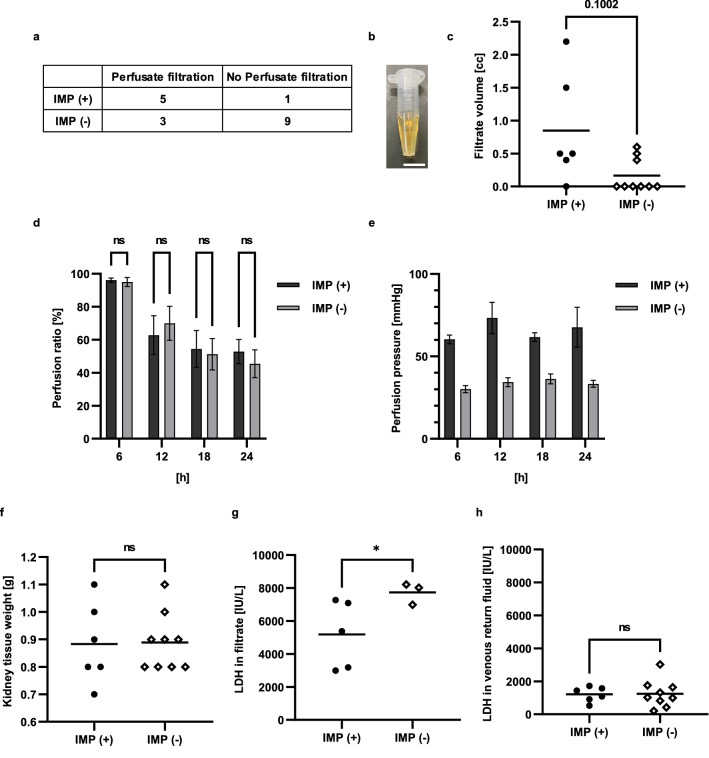
Effect of 24-h intermittent pressurization on kidney perfusion. (**a**) Filtrate breakdown in a total of 18 perfusion experiments. IMP (+) group: 6 cases, IMP (−) group: 12 cases. Fisher’s exact test: *p* < 0.05. (**b**) The 24-h filtrate collection. Scale bars: 1 cm. (**c**) Comparing filtrate output for 24 h of perfusion. IMP (+) group: 5 cases, IMP (−) group: 3 cases. *P* = 0.1002. (**d**) Kidney perfusion ratio was measured every 6 h for 24 h. The data are shown as means ± standard error of the mean. IMP (+) group: 6 cases, IMP (−) group: 12 cases. (**e**) The mean kidney perfusion pressure was measured every 6 h for 24 h. IMP (+) group: 6 cases, IMP (−) group: 12 cases. (**f**) The difference in kidney tissue weights before and after perfusion was measured. IMP (+) group: 6 cases, IMP (−) group: 9 cases. (**g**) LDH was analyzed for 24 h of filtrate collection. IMP (+) group: 5 cases, IMP (−) group: 3 cases. *p < 0.05. (**h**) LDH was analyzed for the perfusing solution leaving the venous outlet tube at 23–24 h. IMP (+) group: 6 cases, IMP (−) group: 9 cases.

Following the findings of the short perfusion experiment, where IMP during perfusion aided uniform circulation in the kidney, we investigated the effect of IMP during 24-h perfusion. The number of samples with no filtrate (6) and samples where the perfusing solutions returning via the venous outlet tube stopped (3) accounted for 75% (9 of 12) of all samples in the IMP (−) group, according to the perfusion studies in 18 cases. On the other hand, samples with no filtrate (1) and perfusion failure (0) in the IMP (+) group was around 17% (1 of 6) (Fisher's exact test: *P* < 0.05; Fig. 4a).

Filtrate was collected for 24-h perfusion (Fig. 4b), and the volume of filtrate tended to be higher in the IMP (+) group, compared with the IMP (−) group (IMP (+): 0.85 cc vs. IMP (−): 0.16 cc, *P* = 0.10; Fig. 4c). Devices in the system monitored the perfusion ratio and pressure during the 24-h perfusion. The perfusion ratio revealed no differences in variability between the IMP (+) and IMP (−) groups over time. The mean perfusion ratio for 24 h was 66.53% in the IMP (+) group and 65.41% in the IMP (−) group (Fig. 4d). The mean perfusion pressure in the IMP (+) group was 65.75 mmHg and 33.53 mmHg in the IMP (−) group (Fig. 4e). The difference in kidney tissue weights before and after perfusion was measured to evaluate cell edema, and both groups averaged + 0.88 g. (Fig. 4f).

As a marker of tissue injury, we measured lactate dehydrogenase (LDH), a deviating enzyme, in 24-h filtrate and venous return fluid at 23–24 h. The IMP (+) group averaged 5182 IU/L in 24-h filtrate collections, while the IMP (−) group averaged 7742 IU/L (*P* < 0.05; Fig. 4g). Furthermore, the IMP (+) group averaged 1208 IU/L in venous return fluid at 23–24 h, and the IMP (−) group averaged 1244 IU/L (Fig. 4h). These results indicate that IMP during perfusion preserved the ability to filter perfusate for 24 h and resulted in less cellular damage than the group without pressurization in terms of filtrate LDH levels.

### Renal histology and detection of remaining cell nuclei after 24-h perfusion

**Figure 5:**
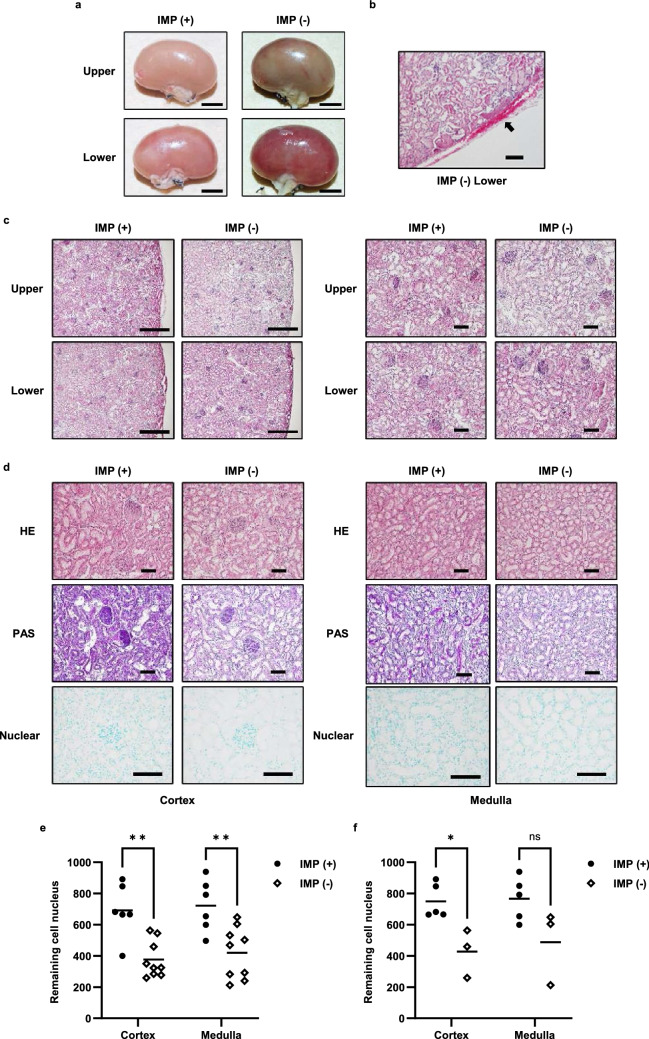
Renal histology and detection of remaining cell nuclei after 24 h of perfusion. (**a**) Tissues after 24-h perfusion of the upper side and lower side. Representative examples from the IMP (+) and IMP (−) groups are shown. Scale bars: 5 mm. (**b)** H&E staining histologic images of kidney sections from the lower side. Black arrows: blood remaining between the parenchyma and the capsule. Right images scale bars: 100 μm. (**c**) H&E staining histologic images of kidney sections from the upper and lower sides. Representative examples from the IMP (+) and IMP (−) groups are shown. Left images scale bars: 500 μm. Right images scale bars: 100 μm. (**d**) H&E, PAS, and methyl green staining histologic images of kidney sections from the cortex and medulla. Representative examples from the IMP (+) and IMP (−) groups are shown. Scale bars: 100 μm. (**e**) The number of nuclei remaining in the stained images. Five sections in the cortex and three sections in the medulla were analyze and averaged. IMP (+) group: 6 cases, IMP (−) group: 9 cases. **p < 0.01. (**f**) The number of nuclei remaining in the stained images of samples with filtrate output during the 24 h of perfusion. IMP (+) group: 5 cases, IMP (−) group: 3 cases. *p < 0.05.

The coloration of the kidney tissue in the IMP (−) group after 24-h perfusion was different from the IMP (+) group in the upper and lower sides of the tissue (Fig. 5a). Pooled blood was observed between the parenchyma and lower side of the capsule in the IMP (−) group (Fig. 5b). Hematoxylin and eosin (H&E) staining revealed that the loss of parenchyma cells in the upper and lower sides of the kidney tissue after perfusion in the IMP (+) group was similar, whereas the upper side of the IMP (−) group showed a more obvious loss of parenchyma cells than the lower side (Fig. 5c). Comparing the cortical and medullary parts of the central kidney tissue stained with H&E to the structure of the IMP (+) group, the histological structure of the IMP (−) group showed atrophy and loss of cells, irregular tubule dilation, and expansion of interstitial space (Fig. 5d). When comparing the brush border in the IMP (−) and IMP (+) groups, periodic acid-Schiff (PAS) staining revealed that the brush border in the IMP (−) group was decreased and absent (Fig. 5d). On the other hand, based on the findings of H&E staining, the IMP (+) group displayed a lesser degree of cell atrophy and loss than the IMP (−) group, and the borders between cells were more distinct (Fig. 5d). According to the PAS staining findings, the brush border was more preserved in the IMP (+) group than in the IMP (−) group (Fig. 5d). Furthermore, nuclear staining results revealed more nuclei in the IMP (+) group than in the IMP (−) group, both in the cortical and medullary regions (Fig. 5d). Analytical software was used to count stained nuclei, and it was found that the IMP (+) group exhibited significantly more nuclei in both the cortical (IMP (+): 691.4 vs. IMP (−): 376.1, *P* < 0.01; Fig. 5e) and medullary (IMP (+): 721.6 vs. IMP (−): 420.4, *P* < 0.01; Fig. 5e) regions. In addition, the analysis of samples with filtrate output followed the same pattern as the results of the total samples, with considerably more nuclei remaining in the cortical area (IMP (+): 749.7 vs. IMP (−): 427.1, *P* < 0.05; Fig. 5f). In both groups, samples with filtrate output had more nuclei than samples without filtrate output. These findings revealed that IMP during perfusion maintained uniform circulation in the kidney and assisted upper side circulation in reducing histological damage, such as cell atrophy and desquamation, as well as inhibiting loss of nuclei caused by cell death.

## Discussion

In this study, we hypothesized that IMP could maintain healthy tissues and circulatory balance when organs were perfused ex vivo. To test this hypothesis, we performed a perfusion culture of rat kidneys at physiological temperature and evaluated the circulatory distribution in the kidney, tissue morphology changes due to injury, and perfusate filtration. The IMP (−) group showed significantly poorer circulation on the upper side than on the lower side, as well as atrophy, cell desquamation, irregular tubule dilatation, interstitial opening, and brush border reduction or loss, all of which were histologically confirmed. On the other hand, circulation increased on the upper side in the IMP (+) group, and the histological damages were reduced. This suggested that the IMP (−) group renal tissue had suffered considerable internal damage. Furthermore, the IMP (−) group produced almost no filtrate, whereas the IMP (+) group retained perfusate filtration ability for 24 h. These results indicate that pressurization to ensure a consistent supply of the perfusing solutions to all parts of the organ helps in organ preservation ex vivo.

The necessity of pulsatile flow while artificially perfusing isolated organs via circulation has long been the subject of research. Luke et al.^23^ compared the histology of post-perfusion kidneys with pulsatile and constant flow during ex vivo kidney perfusion and found no discernible difference in outcomes. In addition, there were no significant differences in the levels of NGAL-1, a measure of kidney injury, and IL-6, a marker of inflammation, in the filtrate. Thus, they found no difference in tissue damage caused by pulsating and non-pulsating flow. On the other hand, Gallinat et al.^24^ demonstrated that pulsatile flow improves sodium reabsorption and creatinine clearance by increasing vasorelaxant NO and decreasing vasoconstrictor ET-1, compared with steady flow. Additionally, pulsatile flow decreased the release of LFABP, a marker of proximal tubular cell damage, into the filtrate. Therefore, it is controversial whether pulsatile flow lessens organ damage when perfusing organs ex vivo. In this study, pulsatile flow was produced through external pressurization, and it lessened tissue damage in the post-perfusion kidney, compared with steady flow. In particular, we saw that pulsatile flow suppressed irregular proximal tubule dilation and brush border reduction or loss in proximal tubular cells. This is consistent with the results showing that pulsatile flow caused by intravascular pressure loading suppressed damage to proximal tubule cells. Furthermore, when perfusion was conducted at a steady flow, residual blood remained on the lower side of the kidney, which led to perfusion failure, and the perfusing solutions occasionally became stagnant; however, this did not happen when perfusion was conducted at a pulsatile flow. This suggested that there was differential tissue damage on the upper and lower sides. Therefore, compared with steady flow, pulsatile flow is expected to have a positive impact on the release of NO and ET-1, which are involved in controlling the internal pressure of blood vessels and preventing blood flow interruption or congestion. This would maintain the volume of circulation in organs and minimize tissue damage.

Instead of widening the vessel lumen with a pump, we achieved pulsatile flow by increasing its internal pressure via external pressurization. Because pulsatile flow generated by a pump increases pressure inside the blood vessel due to the increased flow volume, excessive pressure stress may damage vascular endothelial cells and disrupt the vascular structure. Furthermore, when the difference between internal and external pressure rises with the increase in intravascular pressure, the perfusing solutions leak from the ruptured regions of the vascular structure. Furthermore, when the lumen of a vessel is blocked, the blockage is difficult to open using only the upstream pulsatile flow generated by the pump, and the perfusate may not circulate uniformly in the organ. On the other hand, pulsatile flow generated by external pressurization does not dilate the vascular lumen, which may prevent vascular structure breakdown due to pressure stress. In addition, external pressure suppresses perfusing solution leakage from the rupture area by reducing the difference between intravascular and external pressure. Furthermore, the lumen diameter at the back and forth of the occluded area can be changed by applying external pressure to the whole organ in addition to the upstream perfusion pressure, which is expected to reduce the risk of blood vessel blockage. This principle is also used to prevent venous thrombosis in the deep lower extremities via intermittent pneumatic compression^25,26^. Physical lower extremity stimulation through external positive pressurization acts on the entire vascular system and increases circulation by changing the cross-sectional area of the blood vessels^27^. The luminal diameter is decreased by pneumatic compression, which speeds up blood flow and prevents venous thrombus development. When blood flow is congested, pneumatic compression also aids in opening and closing venous valves that have lost the ability to act as non-return valves, reducing blood backflow and alleviating blood flow congestion. In addition, the increase in interstitial pressure around the capillaries caused by pneumatic compression decreases water exudation from the capillaries and maintains microvascular blood flow. This improvement in blood flow congestion and suppression of extravascular water exudation can be seen as a shared characteristic between the intermittent pneumatic compression technique employed in clinical practice and the external pressurization we carried out during organ perfusion. In this study, intermittent external pressurization was also involved in residual blood drainage inside the kidney at the start of perfusion. Visualizing red blood cell migration in the perfusing solutions using a blood flow imaging system showed that perfusing solutions containing blood components flowed out of the kidney toward the renal vein tube in intermittent changes (Supplementary Movies 1, 2). Increased residual blood drainage is believed to decrease the danger of vascular occlusion and is most likely the result of a temporary increase in flow speed due to pressurization.

This study showed no difference in perfusion ratio between the IMP (+) and IMP (−) groups. This may be the result of the following two factors. The first, the kidney vascular ends do not have cuts, where the entire organ was harvested, and perfusing solutions do not leak directly out of the kidney, unless the blood vessel structure and function are compromised. Therefore, it is inferred that there was not much difference in perfusion ratio with or without external pressurization. The second reason for the lack of difference in perfusion ratio is the effect of albumin addition to the perfusing solutions. The main function of albumin in vivo is to retain water in the blood vessels by maintaining its osmotic pressure and thereby preserving normal blood circulation. As albumin in the blood decreases, osmotic pressure drops, and water exudes into the extravascular interstitium, which results in reduced blood volume in the vessels. This causes symptoms such as edema and decreased blood pressure. When albumin is added to the perfusate, and organs are perfused ex vivo, reduced interstitial edema has been reported^28^. Because albumin was added from the beginning to increase colloid osmotic pressure, the IMP (−) group in this study is also estimated to have suppressed exudation to some extent. Therefore, there was no significant difference in perfusion ratio with or without external pressurization and in kidney tissue weights before and after perfusion to evaluate cell edema. For these two reasons, the present kidney perfusion seems not to have led to a difference in perfusion ratio.

As described above, there was no difference in perfusion ratio between the IMP (+) and IMP (−) groups in this study, but circulatory distribution in IMP (+) group kidneys improved, compared to that of the IMP (−) group. This may be caused by intravascular pressure during perfusion. The mean perfusion pressure in the IMP (−) group in this study was 33.5 mmHg, which was considerably lower than the intravascular pressure of 60 mmHg in the glomerular artery in vivo, as shown in Fig. 4e. On the other hand, the mean perfusion pressure in the IMP (+) group was 65.7 mmHg. As Patel et al.^29^ reported, kidneys perfused at 75 mmHg during normothermic machine perfusion produced approximately five times as much filtrate as those perfused at 55 mmHg; therefore, setting a higher mean perfusion pressure may have improved circulatory distribution within the kidney in this study. Glomerular capillary pressure must be at least 60 mmHg to filter perfusate. Lower intravascular pressure is less than the sum of colloid osmotic pressure and the internal Bowman's capsule pressure, so there is not enough pressure to induce hemofiltration to the tubular cells, and perfusing solutions are not filtered. Therefore, the IMP (−) group had lower mean perfusion pressure and more samples with no filtrate output. On the other hand, the IMP (+) group had more samples with filtrate output than the IMP (−) group because sufficient mean blood pressure was ensured, which indicated that the ability to filter perfusate was maintained by intermittent external pressurization during 24 h of normothermic perfusion. Therefore, perfusion throughout the kidney using adequate pressure and flow rate due to external pressurization in the IMP (+) group is considered to have resulted in increased filtrate volume due to increased glomerular filtration rate, compared with the IMP (−) group.

On the other hand, perfusion pressure does not necessarily have to be high to maintain circulation in the organs. Excessive stress caused by increased perfusion pressure may injure vascular endothelial cells, possibly disrupting vascular structure and damaging tissues. Urcuyo et al.^30^ reported that whole blood increased intravascular pressure during normothermic machine perfusion of porcine kidneys and caused glomerular necrosis, compared with storage solutions. Srivastava et al.^31^ also analyzed the relationship between the cells constituting the glomerulus and the fluid shear stress. They reported that glomerular epithelial cells are very sensitive to shear stress, which may induce detachment and damage these cells. We preliminary made a comparative of the flow rates. The perfusion at 100 μL/min showed a higher decrease in perfusion ratio as compared to the perfusion at 50 μL/min, which was the flow rate set in this experiment (Supplementary Data 3). This result suggested that increasing the flow rate, i.e. increasing the perfusion pressure, might further decrease the perfusion ratio. Therefore, perfusion pressure is an important factor in maintaining adequate circulation when the organ is perfused ex vivo, but excessively high pressure may result in tissue damage and inadequate circulation in the organ. Hence, an external pressure of + 100 mmHg was used in this experiment to keep the perfusion pressure within the range of 120 mmHg as the upper limit of normal biological blood pressure. The mean perfusion pressure in this study was 65.7 mmHg, comparable to that in vivo. This is considered a suitable condition to avoid tissue damage caused by decreased circulation volume due to low pressure and excessive stress due to high pressure.

Ink was used to mark the glomeruli, through which the perfusing solutions passed, thus evaluating kidney circulation. Ink particles (approximately 150 nm) accumulated in the glomerulus without passing through the filtration barrier, because the three-layered glomerulus filtration barrier is approximately 40 nm. We previously reported that circulatory distribution is biased towards the lower side in small intestinal tissues in perfusion experiments without pressurization^20^. Similar to this report, circulation regions in kidney perfusion in our study were also biased towards the lower side. When tissues and organs are put in the ex vivo perfusing chamber, an imbalance in circulation occurs regardless of the perfusion object, biasing the perfusate towards the lower side due to gravity. On the contrary, a mechanism is provided in vivo to ensure that blood circulates uniformly throughout the organs. For example, blood is delivered evenly to the organs through feedback regulation of circulating volume and vascular resistance via the autonomic network^32^. As a comparison, the number of glomeruli with ink accumulation was analyzed when the ink was delivered from the abdominal aorta to rat kidneys, and the results showed that ink was accumulated in approximately 90% of the glomeruli (Supplementary Data 4). On the other hand, when applying external pressurization ex vivo, the ink accumulated in approximately 60% of the glomeruli. The results showed that in vivo circulation was more uniform than in vitro circulation. Therefore, external pressurization considerably improved kidney circulation under ex vivo perfusion, but a further examination of circulating volume feedback control and vascular resistance is needed to mimic the circulatory distribution to that in vivo.

Acute tubular necrosis, an ischemic lesion, is morphologically characterized by tubular epithelial cell damage and necrosis. The main lesions include irregular tubule dilation, epithelial cell flattening and detachment, and brush border reduction or loss. Previous studies on kidney transplantation have reported that tubular cell apoptosis had already begun during the 1-h cold ischemia of the kidney^33,34^. Widespread tubular cell damage was reported afterwards, including brush border reduction or loss. In a study analyzing changes in tubular cells during 6 h of cold storage, destruction of cell structure was observed after 3 h of storage^35^. Additionally, the research team reported nuclear aggregation and nuclear fragmentation (disintegration) as damage processes, and nuclear changes could take place even in an ischemic state lasting just 1 h. Here, the brush border was also reduced or lost in the PAS staining images 24 h after perfusion in both the IMP (+) and IMP (−) groups. The process of cell death in injured cells results in nuclear aggregation and fragmentation, which may explain why fewer nuclei persisted in the IMP (−) group than in the IMP (+) group. We consider that proximal tubular cell deterioration and necrosis have spread, as in acute tubular necrosis. The brush border in the IMP (+) group was remained, compared with the IMP (−) group, because external pressurization prevented erythrocyte plugging and ensured peripheral circulation from the early stage of perfusion. Thus, we consider that the reduction in the hypoperfusion regions reduced early tubular cell damage and delayed its progression by improving perfusion balance.

In this study, we concentrated on developing a perfusion method that permits uniform circulation and blood vessel protection, both of which are crucial for ex vivo organ perfusion. To this end, oxygen concentration is a factor that improves organ perfusion conditions. Although the experiments in this study did not consider the perfusate oxygen concentration, reports analyzing the effects of hypoxia on proximal tubular cells have shown significant disruption of tissue structure, such as cell expansion and changes of the brush border^36^. In addition, oxygenation reportedly improves aerobic metabolism and inhibits tubular cell damage^37^. Therefore, we also consider that the effects of oxygen deficiency should be taken into account. Currently, the use of blood^38–41^, artificial oxygen carriers^42^, and metabolism control via temperature regulation^30,43^ are being investigated to cope with oxygen consumption under physiological conditions where metabolism is actively progressing. We expect that using this strategy in combination with an external pressurization system could result in longer-term organ preservation ex vivo.

The evidence provided in this study showed that the intermittent pressure approach greatly reduced the problems associated with perfusion imbalance during ex vivo tissue and organ perfusion, thus, keeping tissues healthy. This method is expected to preserve organs more efficiently in transplantation medicine. This might then result in fewer organs being discarded and more organs being made available for transplantation. In the field of regenerative medicine, regenerating tissue that can be perfused ex vivo is increasing in prevalence. This method may help construct tissues with functions similar to those in vivo by supplying oxygen and nutrients to every corner of the tissue and removing accumulated waste during the ex vivo perfusion of these regenerated tissues. Therefore, this IMP approach is expected to contribute meaningfully to transplantation and regenerative medicine.

## Materials and methods

### Ethics statement

All animal experiments were performed following the guidelines of the Ethics Committee for Animal Experimentation of Tokyo Women’s Medical University and in compliance with the Legislation and Regulation on the use of animals in biological research with the ARRIVE guidelines. All experimental protocols were approved by the Ethics Committee for Animal Experimentation of Tokyo Women’s Medical University. All animals were housed in individual cages with free access to food and water under a light/dark cycle of 12 h and maintained at constant room temperature and humidity. Animals were euthanized by exsanguination under 5% isoflurane in accordance with the American Veterinary Medical Association (AVMA) euthanasia guidelines.

### Animals

Sprague–Dawley rats (aged 8–11 weeks; male) were obtained from Japan SLC, Inc., Japan. This study was carried out on kidneys excised from rats weighing 250–300 g.

### Kidney excision and perfusion culture preparations

Since anatomical issues made the removal of the right kidney and cannulation technically difficult and likely to interfere with the experiment, only the left kidneys were used for purpose of this study. The animals were anesthetized with 2–3% inhaled isoflurane. The fatty tissue around the left kidney was removed after median abdominal incision. For cannulation, the arteriovenous and ureter were exposed and isolated. Heparin (400 IU/kg) was administered via the penile vein, followed by a 2-min wait. Then, papaverine hydrochloride (16 μL/kg) was administered via the penile vein, and the left kidney was removed after 5 min. After appropriate length resection of the renal arteriovenous, the kidney tissue was gently washed 5 times with Hanks salt with 1% penicillin–streptomycin and 0.9 μg/mL papaverine hydrochloride. The left kidney with arteriovenous was implanted in a custom-made sealed chamber and connected to the designated tubes for the artery, vein, and ureter (Fig. 2a). Solutions were perfused from the artery at 50 μL/min using the normothermic machine perfusion system (Fig. 2b).

### Normothermic machine perfusion system

The excised kidneys were perfused ex vivo by using the normothermic machine perfusion system consisting of the following elements: perfusion pump (Ismatec Co., Ltd., Germany), temperature control unit (Tokaihit Co., Ltd., Japan), custom-made sealed chamber for the left rat kidney (Tokaihit Co., Ltd., Japan), custom-made cannulation tube for rat kidneys (Tokaihit Co., Ltd., Japan), rodent tail vein catheter (Braintree Scientific, Inc., US), flow path tube [fluoro-rubber tube (KING WORKS Co., Ltd., Japan), thermoplastic-elastomer tube (Saint-Gobain Co., Ltd., Japan)], pressure transducer (Edwards Lifesciences Co., Ltd., USA), electrical scale (A&D Co., Ltd., Japan), reservoir bottle for the perfusing solution, waste fluid bottle, and digital microscope camera (AnMo Electronics Corp., Taiwan) (Fig. 2b). The temperature inside the chamber was maintained at 37 °C ± 0.5 °C. The perfusing solutions in the reservoir bottle (4 °C) were infused at the rate of 50 μL/min. The perfusion pressure (arterial pressure) and internal chamber pressure were monitored continuously.

### IMP system

In this study, we devised a new perfusion method that improved upon the mechanisms of the previous system^20^. The previous mechanism relied on a pump to pressurize the chamber by drawing in air, which limited the applicable pressure value and accuracy. The new system (Fig. 1a) pressurized the inside of the chamber by sending air back and forth between the chamber and the inside of the sealed container without drawing in outside air. A wireless logger (TSW-02WR) (Toho Electronics Co., Ltd., Japan) was used to monitor the humidity inside the chamber. An external positive pressure of 100 mmHg was applied for 20 s at 20-s intervals.

### Perfusing solutions

The perfusing solutions consisted of Leibovitz’s L-15 Medium with L-glutamine, phenol red, and sodium pyruvate (FUJIFILM Wako Pure Chemical Corp, Japan) containing 10% fetal bovine serum (Japan Bio Serum Co. Ltd., Japan), 1% penicillin–streptomycin (Life Technologies Co. Ltd., USA), 0.9 μg/mL papaverine hydrochloride (Nichi-Iko Pharmaceutical Co., Ltd., Japan), 20 ng/mL rat FGF-2 (PeproTech, Inc., NJ), and 1.6 g/dL albumin (FUJIFILM Wako Pure Chemical Corp, Japan). A volume of 72 mL/day was prepared for pouring into the chamber at a rate of 50 μL/min. The perfusion circuits were primed before perfusing. In this study, the perfusing medium was designed for atmosphere culture and contained Hank’s balanced salt as a pH buffer solution.

### Short-term perfusion protocols

Short-term perfusion was used to evaluate circulatory distribution. After perfusion pressure was stabilized at around 30 mmHg (tens of seconds), the kidney was perfused with the solutions for 15 min at a rate of 50 μL/min. Finally, ink (2 × dilution) was perfused for 5 min. The ink was used to identify the areas where the perfusing solutions flowed by accumulating ink particles on the glomerulus filtration barrier. IMP was used for 20 min (during perfusion of the solutions and ink). The IMP (+) and IMP (−) groups were compared in each of the five cases.

### Intrarenal circulation analyses

The percentage of glomeruli that had accumulated ink was used to evaluate the perfusion distribution. The IMP (+) and IMP (−) groups were divided into three sections. To detect black particles and nuclei, the sections were stained with PAS using a protocol that did not stain the nuclei. A microscope (BZ-9000, Keyence, Japan) was used to image the stained sections. Glomeruli that had accumulated ink were counted in the stained image. The mean value of the percentage of glomeruli with ink accumulation was calculated. Ink accumulation was defined as the presence of even a few ink particles in the glomerulus.

### Twenty-four-hour perfusion protocols

Twenty-four-hour perfusion was performed to compare the effects of IMP, and changes in tissue morphology due to injury and kidney function were analyzed. IMP was initiated after perfusion commenced and pressure was stabilized, as in the short-term perfusion protocols. Six cases in the IMP (+) group and 12 cases in the IMP (−) group were included. The perfusion ratio was calculated as the volume of perfusing solutions returned via the venous outlet tube divided by the volume of perfusing solutions infused via the arterial inlet tube. The mean perfusion pressure was used to calculate the perfusion pressure for the IMP (+) group. Systolic perfusion pressure was defined as the pressure that increased as a result of pressurization, and diastolic perfusion pressure was defined as the pressure that decreased as a result of depressurization. Then, [(systolic perfusion pressure − diastolic perfusion pressure)/3 + diastolic perfusion pressure] was used to calculate the mean perfusion pressure. Kidney tissue weight was measured before and after perfusion started.

### Histology

The kidneys were fixed with 4% paraformaldehyde (FUJIFILM Wako Pure Chemical Corp, Japan), and 8 μm-thick sections were prepared after paraffin block preparation. The sections were stained with H&E and PAS following standard protocols. Methyl green was used for nuclear staining. The stained sections were imaged using a microscope (Eclipse E800, Nikon, Tokyo).

### Quantitation of remaining cell nuclei

The number of nuclei remaining in the images was counted to evaluate tissue damage. The number of remaining nuclei was measured using ImageJ software. The images were processed via binarization, and the watershed method was applied to separate adjacent nuclei for detection. Five sections in the cortex and 3 sections in the medulla were analyzed and averaged. In the cortex, 1 glomerulus was positioned in the center of the image.

### Analyses of perfusing solution returning via the ureter and vein

LDH was examined (Oriental Yeast Co. Ltd., Japan) to assess 24-h filtrate and the perfusing solutions returned from the renal venous site between 23 and 24 h.

### Statistical analysis

GraphPad Prism 9 (GraphPad Software Inc., USA) was used for statistical analysis. All data were expressed as means ± standard deviation. A Welch’s t-test was performed to compare the IMP (+) and IMP (−) groups to test the difference in means between the data, assuming that the population variances between the two groups cannot be equal. Fisher’s exact test was applied to estimate whether the application of IMP and perfusate filtration are independent of each other. Two Way ANOVA was applied to analyze the quantitative data for perfusion ratio and cell nucleus. A *P* value < 0.05 was considered significant.

## Supplementary Information

Supplementary Video 1.

Supplementary Video 2.

Supplementary Information 1.

Supplementary Information 2.

Supplementary Information 3.

## Data Availability

The data that support the findings of this study are available from the corresponding author upon reasonable request.
