# Supplementary material for: External pressure dynamics promote kidney viability and perfusate filtration during ex vivo kidney perfusion
Source: Sci Rep. 2022 Dec 13;12:21564. doi: 10.1038/s41598-022-26147-5 (PMC9747902; doi:10.1038/s41598-022-26147-5)
Supplement: Supplementary file 3 — Supplementary Information 1. [file 41598_2022_26147_MOESM3_ESM.docx]

**Supplemental data**

**1.**

Residual blood drainage inside the kidney with the first intermittent pressurization after the start of perfusion.

**2.**

Perfusate outflow including blood components from inside the kidney to the venous outlet tube with intermittent pressurization for short-term perfusion. The change in wash-out of blood remnants induced by intermittent pressurization was monitored using laser Doppler blood flow assessment: moorFLPI-2 (Moor Instruments, UK). Time-series of pseudo-color processed images of blood flow tissue oxyhemoglobin and deoxyhemoglobin concentrations via laser speckle. The images are colored using pseudo-color processing, calculated by the captured relative blood flow distribution.

**3.**

Kidney perfusion ratio comparing the flow rates was measured every 1 h for 4 h. The data are shown as means ± standard error of the mean. 50 μl/min: 4 cases, 100 μl/min: 3 cases.

**4.**

Point diagram comparing the percentage of glomerular counts accumulated by administration of ink via the abdominal aorta *in vivo* (n=3). IMP (+) and IMP (-) group: 5 cases.
