# Supplementary figures and images for: External pressure dynamics promote kidney viability and perfusate filtration during ex vivo kidney perfusion
Source: Sci Rep. 2022 Dec 13;12:21564. doi: 10.1038/s41598-022-26147-5 (PMC9747902; doi:10.1038/s41598-022-26147-5)

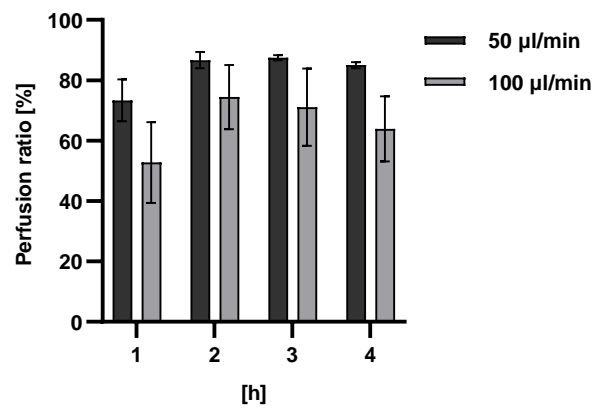

Supplemental data 3

Supplement: Supplementary file 4 — Supplementary Information 2. [file 41598_2022_26147_MOESM4_ESM.pdf]

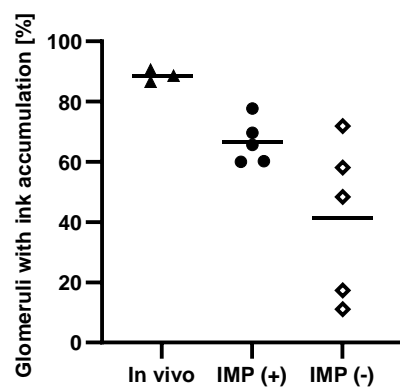

Supplemental data 4

Supplement: Supplementary file 5 — Supplementary Information 3. [file 41598_2022_26147_MOESM5_ESM.pdf]
